# Supplementary material for: Patterns of Intron Gain and Loss in Fungi
Source: PLoS Biol. 2004 Nov 30;2(12):e422. doi: 10.1371/journal.pbio.0020422 (PMC532390; doi:10.1371/journal.pbio.0020422)
Supplement: Table S1 — Also available at http://genes.mit.edu/NielsenEtAl/. (4.3 MB ZIP). [file pbio.0020422.st001.zip › NielsenEtAl/html/270.html]

AN5109.1.NCU01759.1.MG03160.1.FG06240.1


```
 CLUSTAL W (1.82) Multiple Sequence Alignments - Introns Inserted


Sequence 1: AN5109.1	294 aa
Sequence 2: FG06240.1	304 aa
Sequence 3: MG03160.1	311 aa
Sequence 4: NCU01759.1	322 aa
Alignment Length: 337 aa
Number Identitical Residues: 105 aa
Alignment Score (without introns) 6035


MG03160.1 	MNSAASSHQPDASGEAPKQLN-YLPYLKLNDGNQIP~---------------M0LSYGLG
NCU01759.1	MNSATSNFEVDATGVSPKQIHNLIPSLKLSDGNEIP~---------------M0LGYGLG
FG06240.1 	MSSHN------------------IPSYKLNDGNEIP0NSQHTAALSHKANICK~LAFGLG
AN5109.1  	MVPTS------------------IPTTQLKDGTSIP~---------------V0IGYGTG
          	* .                    :*  :*.**..**                  :.:* *

MG03160.1 	TAQCRR-GGD--VDPKLVELTKIALKKGYNHLDGAEV1YGNEEELGQAVKESGLPRESLF
NCU01759.1	TANYKSGDSADTISEPIVNATLTALNAGYAHLDGAEV~YGNEVELGRAIAQSKLPRSSLY
FG06240.1 	TAQFKK-DSQSGLDQHVVDITTKAIKAGY-------L1YGNEEELGEAIKAGGVPREQLY
AN5109.1  	TAWFKK-KGDTSINRDLVESIKTAIRLGYHHLDSAEV~YGTERELGVAIKECGVPREQLF
          	**  :   .   :.  :*:    *:. **   ..:.: **.* *** *:    :**..*:

MG03160.1 	ITTKTFCKPGVTTQESLDASLKRLQLDYVDL~FLIHSPFWAESPEELQAKWAEMEALREA
NCU01759.1	ITTKTIVRAGDTVTQSFNRSLEKLGLDYVDL~YLLHSPFFAKNPEQLQAAWREMEKIKDS
FG06240.1 	VTTKIPAEKKGSAIDAFDVSLKKLGLEYVDL~YLIHGPWFAETDEDLQQRWSELEQIKES
AN5109.1  	VTTKVNQN-IANVPKALEDSLEKLQLSYVDL2YLIHQPFFAESPTELQDAWAAMEKVKEA
          	:***   .   .. .::: **::* *.**** :*:* *::*:.  :**  *  :* ::::

MG03160.1 	GKAKSIGVSNFLQEHLETILKTA---KVPPAINQIEYHPYLQHG----DLLDYHRKQNIA
NCU01759.1	GMAKSIGVSNFLISHLQTLFDIPGGLQYPPVVNQIEYHPYLQHVVEGEDLVAFCREKHIA
FG06240.1 	GRAKSIGVSNFLQVHLEPILKTA---KVKPAINQIEYHPYLQHG----DLLDFHKKHNIA
AN5109.1  	GKARAIGVSNFLESHLETILDSA---RIPPAINQIEYHPYLQHG----SLVPYHERKGIA
          	* *::*******  **:.::. .   :  *.:***********     .*: : ..: **

MG03160.1 	TSAYGPLTAITNAKGGPVDGKYHELARKYGVTPGEIALRWCIDQGVVAITTSAKEDRLEA
NCU01759.1	IAAYSPLTAITKARPGPVDPIYTQLAKKYGVSEAEIAFRWCLDQGIVTLTTSSNEERLKG
FG06240.1 	VSAYGPLTPIVTAKGGPVDPLYTKLAEKYGVTESEVGLRWVIDQDTIALTTSNKVERLDG
AN5109.1  	VASYGPLTPVTRAKGGPLDPLLSELAGKYGVGEGEILLRWSLYRGAVAITTSGKESRLST
          	 ::*.***.:. *: **:*    :** ****  .*: :** : :. :::*** : .**. 

MG03160.1 	LQKRIPSFKLTPKEVQEISELGNQKHHRGFWNNKFAADDRR
NCU01759.1	YLKKLPSFKLTPKEVEDIASAGKEKHYRVWWTEEFAGDDRH
FG06240.1 	YLAKLPKFKLTQDEIAEISKLGAEKHFRGFWRNKFDENDRS
AN5109.1  	YLN-VFKFQLTHEEVLKISRIGDQKHYRGFWSDKFAADDRS
          	    : .*:** .*: .*:  * :**.* :* ::*  :**
```
